# Practical Experience in Typhoid Fever as It Occurs in Alabama

**Published:** 1888-08

**Authors:** Frank Prince

**Affiliations:** Bessemer, Ala., Ex-Vice-President and Senior Counselor of the Medical Association of the State of Alabama


### PRACTICAL EXPERIENCE IN TYPHOID FEVER AS IT OCCURS IN ALABAMA.

BY FRANK PRINCE, M. D., BESSEMER, ALA.,

Ex-Vice-President and Senior Counselor of the Medical Association of the State of Alabama.

CONCLUDED.

Just here I desire to refer to a letter of Dr. Benjamin H. Riggs, addressed to Professor P. H. Mell, Jr., Auburn, Alabama, in which he says: "I am studying to account for an unusual healthfulness in this vicinity for the past four months or longer. It has seemed that we have had more rainfall, lower temperature and more frequent thunder-storms. Perhaps the dilution of our drinking water, the washing of the atmosphere and the ground surface, and the absence of long-continued high temperature, have been the main factors." To these excessive rainfalls, as delineated by him, in connection with some other factors, are we disposed to attribute a large amount of typhoid fever, of a virulent type, in township 18, 19 and 20, range 45 and 46. The sudden changes from cold to hot, or the rise and fall in temperature, exerts a great influence upon the system.

If we rely upon the germ as the sole cause of disease, then the quantity of rainfall, the change of temperature, nor the dilution of drinking water, would be of any consequence if the germ was not there.

It has not been satisfactorily demonstrated, with all the microscopical investigations and cultured field experiments, that the germ was not the product of the disease. The fact of its existence does not demonstrate that it originated the disease, but the fact from the observations pursued thus far, that each disease has its germ, advances the idea at least that each disease generates its own germ. As far removed as I am from these fields of experiment, you might say to me that "fools enter where angels fear to tread." But the world is made up of fools, and from them thought evolves thought that sometimes generate the greatest scientific achievements, and the fool of to-day may be the hero of another cycle. I am unwilling to believe that the germs that infest the human body in disease are always the product of the vegetable world, or of other influences exterior to the body, but often the development from or of the body itself when acted upon. A cell is a morphological unit and enters into the formation of every plant and animal, and occupies a distinct individuality in all organism. One of the earliest revelations of the microscope was the myriads of minute living specks in the water or mud where decaying animal or vegetable matter had been infused. By many these specks are regarded as *de novo* productions from the decaying cells, while by others they are ascribed to germs that pervade the atmosphere in countless numbers and everywhere, and possess an almost indestructible tenacity of life. Whether they originate in the death of a cell, or whether from the atmosphere without, there can be no doubt but that they play an important part in the causation of diseases. It is quite conceivable that different species of organism in the system, by its death, may produce different compounds of vital, active, deleterious nature, with special affinities for various organs, and thus produce a train of symptoms *sui generis.* Such, for instance, as ptomaines, the noxious alkaloids found after the death of organic tissue.

Take hæmoglobin as an example. This compound has a definite rôle to play in the economy, but it can only do its work when contained in the red blood corpuscle. Let it escape and float free in the blood, it not only becomes useless, but sets up a violent irritation, if not a distinct inflammation, in the process of elimination from the kidney. Then take the salts of potash when these are set free in the blood, according to Bernard and Grandeau, results in general nervous depression and paralysis of the muscular fibres of the heart. Then the salts of iron, and lime, and magnesia, which enter normally into the composition of one or other of the tissues when set free in the blood, are poisonous, and bring about, to say the least of it, abnormal condition of otherwise healthy organic tissue. Then take these and kindred anatomical and physiological facts, and it would demonstrate that disease does not always originate from germs exterior to the system, or that germs are not the sole cause of disease.

### RESULTS OR COMPLICATIONS.

For the last few years there have been more cases complicated with phlebitis than many years previous, and it often happens that if the first case in a family is attacked with phlebitis, others are most sure to follow. This sometimes is ushered in with a slight shivering sensation and a want of action in the leg. It feels heavy and distended. Pain soon begins, and in many cases becomes severe, almost unbearable. The leg enlarges, often to twice its natural size, and sometimes the swelling is very obstinate, lasting many months before it is entirely relieved. In a few instances that I have known the leg never returned to its natural size, and, although years have passed, still the man carries an enlarged limb, but with no defect in vitality, muscular action, and no pain. In treating this, my plan has always been to elevate the foot to an angle of about 45° on a gradual ascending plane, and apply mullein leaves steeped in hot vinegar, and around this a hot flannel cloth or blanket, closely wrapped to keep all in position, and change as often as they cool. If this does not relieve, then I direct the use of a liniment of: ℞. Oil origanum, ℥j; oil sassafras, ℥j; tincture capsici, ℥jss; aqu ammonia, ℥j; spirits camphor, ℥j; chloroform, ℥jss; well rubbed on the leg, and re-apply the mullein and hot flannel over this. Relief soon follows.

*Parotid abscess.*—I regard this as a relief to the patient, as recovery soon follows. Encourage suppuration and open as soon as possible.

*Convulsions.*—Seldom have I seen complications of this kind, and then only in children. This I attribute to excessive fever heat, and in no instance would they return where cold was freely applied to the head and continued until temperature was reduced. Pyrexia is due to heat production being above normal and its elimination subnormal. Sometimes it is the result of nervous phenomena without any apparent waste of active tissue. Sometimes the nervous system is overcome by the excessive heat, and convulsions ensue.

Then again, as another complication, we have a sluggish, inactive brain. Memory for the time being seems to have been lost, both in names and dates. But this is only a question of time, as with returning health brain reaction takes place.

*Deafness—ulcerated cornea, with temporary loss of vision—suppuration of internal ear.*—I have never known these complications to be permanent; on the contrary, most cases of typhoid fever, after recovery, seem to take on a new lease of life. Fuller habit, or accumulation of adipose tissue, but vigor of constitution, or physical vitality not increased. Loss of hair and loss of toe-nails, the result of long-continued heat and dryness of the capillaries, and sometimes accompanied with this is general denudation, or shedding of the skin.

### TREATMENT.

Good common sense is a prerequisite to successful treatment of any disease, but more especially of typhoid fever. A well-grounded preparation theoretically, as well as therapeutically, is greatly to be desired, and, like a well-ordered household, under the guidance of common sense, will carry peacefully and happily through.

Sometimes you will be enabled to see the patient early in the attack, and if a case of typhoid mitior, or constipated typhoid fever, let the bowels be opened with some gentle aperient. But it more often than otherwise happens that this part of the treatment has been instituted before you see the case. In a case of obstinate constipation I give a pill of calomel, opium and ipecac, repeated till the bowels are acted upon, nitre and turpentine to be given two hours after each pill. If there is no action on the bowels after twenty-four hours, an enema of ginger tea, or molasses and soap and warm water. So soon as the bowels are opened, if there is but little gastritis, I order a continuation of turpentine and nitre, alternating every two hours with carbolic acid in one-drop doses. But if there is much gastric complication, I prefer sulphuric acid to anything else. Rub the bowels well with turpentine, and if the soreness and tympanitis is very great apply a turpentine stupe. To aid in the reduction of the fever, make free use of cold water by sponging and pouring, and, as is the case sometimes, I find it necessary to use ice freely; eat it, beat it up and tie it in a dry towel, and apply to different parts of the body at the same time, and if this will not reduce the temperature below 104°, then give 20 grains of antipyrin, and repeat it in two hours if necessary, and the temperature soon goes down to 100°. But this excessive heat is apt to return in twenty-four hours, when the same treatment, if persisted in, will overcome it, and the temperature in two or three days will be normal, to rise again, but never to extreme heat. I have never seen bad effects from the free, even excessive, use of cold water. It never has in any case brought on collapse, but, on the contrary, it has often prevented it, by relieving the system of the terrible oppression, the result of excessive heat. It is the best tonic we can use in typhoid fever. There is some danger in the use of antipyrin, that is, in increasing gastritis if it exists, or producing it if it does not exist. And it also has a tendency to weaken the action of the heart, which should specially be guarded all through this disease.

By the application of cold water and ice, and ice pack, the supply of oxygen is greatly increased. Oxygen is absolutely necessary in the evolution of nerve force, or vital electricity, and a deprivation will soon result in death.

In these external applications of water, I often add strong apple vinegar.

For days I have kept the feet and legs in a tin basin, where the water was changed as often as it became warm, both day and night, until the fever cooled.

When the discharges are bloody, a pill of opium, calomel and acet. plumbi should be used until all soreness has left the bowels. But there are cases where this will not control, or succeed, and

I use then the dark Pinus Canadensis, alternating the pill, and stop the use of turpentine. The best fever mixture, to be continued all along through the disease, is a teaspoonful of citric acid, and a tablespoonful of the bicarb. potassa, mixed in a goblet of water, and so soon as it is done effervescing give it by the teaspoon, or tablespoonful dose, as the case may require. All along through the attack, I freely use milk punch, of brandy or rye whisky, and milk or egg-nog. There were cases in which I made free use of the iodide of potassium, and with very great benefit to the patient, and if there has ever been any antipyretic with me it is iodide of potassium, and antipyrin itself. Quinine I have always dreaded, and I never use it in this fever. In the hands of others it may be effective, but I have never seen any good from it.

Up to this time, October 22d, this year, there have been, in one section of our county, fifty-three cases of typhoid fever, and I have not given quinine to any one of them, and only one has died, and that one had phthisis.

Opium, in some form, should be used all through the attack, very frequently in the beginning of the attack. I use it in combination with calomel and ipecac. I prefer the gum opium to any other preparation, and especially where there is extensive ulceration, or perforation of the bowels. I never let the patient come from under its influence—sometimes for as long as fifteen days. My object is to keep down the peristalic action of the bowels and permit a healthy reaction to take place. I have seen no bad result follow, even though the bowels have been locked up for fifteen days.

There are cases in which a blister has been very beneficial, and when applied they should be large enough and kept discharging as long as possible.

In cases of a very aggravated type, where your patient, after days of restlessness and delirium, goes into a state of collapse and cannot be relieved by alcohol, ammonia, brandy, whisky, capsicum, or any of the diffusible stimulants, I use a half grain nitrate of silver every two or four hours, as the case may require. And

I have seen very happy results from this combination, in an extremity, when hope seemed almost gone.

No case of typhoid fever should be treated by a regular routine system, or even by what you read in the medical books. Every case has its own individuality, and in all points is not like any other you ever saw. They differ in many points, and it is all-important that symptoms should be treated and the wants of the whole system always in view. Never get behind the case, but always keep ahead and anticipate in treatment.

In excessive hemorrhage from the bowels, the best preparation I have ever used is elixir of ergot in teaspoonful doses, and hypodermically, and alternate with opium and acet. plumbi. In case of perforation of the bowel, which is manifested by a fiery red tongue, perfect rest should be enjoined. Egg emulsion of turpentine, alternating with opium and nitrate of silver, or opium and carbolic acid every two hours, and the turpentine applied to the bowels or abdomen. Where there is much delirium, bromide of potassium or camphor water. Acids of any kind I have always found beneficial in this fever and always agreeable to the patient.

I have delineated a general plan of treatment that I have followed for over thirty-five years with success, and a few words upon diet and I am done. Let no solid food whatever be given. In the great majority of cases, sweet milk is preferable to anything else. But there are many who are better served with buttermilk, or milk after it has been churned. There is a peculiar acid that belongs to this quality of milk, which makes it very acceptable to the patient. But sometimes milk cannot be used on account of the special aversion of the patient; then my plan has been to substitute chicken water or a tender piece of broiled beef or bird to be chewed by the patient, and when all the fluid is extracted to throw out the solid portion.

There is nothing more dangerous to a convalescing patient in this disease than a disregard of strict dietetic rules. No solid food is at all allowable, and not too great a quantity of milk. I have known too much milk to cause death in a few hours, long after the patient had been clear of fever. Extreme hunger, after severe attacks, often takes place, and the patient, though a man or woman of good sense, unless prevented, will produce death by appeasing or satisfying hunger.

As confirming my views a report of a few of my recent cases is given below:

Caleb G., white,—Oct. 1, sick. Oct. 19, called to see him and found a temperature 105°, pulse small, thready, 130 to the minute, delirious, picking at bed clothes, eruption over the whole body and arms, dark thin discharges, tongue pointed and shaky, with red tips and edges and dark brown at the back, white lighter colored to the form—stools very frequent.

Opium, gr. ¼ acet. plumbi, gr. ½, given every four hours, and in two hours carbolic acid, 2 drops after each pill. Milk punch and milk as a diet. If fever should increase then neutral mixture.

Oct. 20. Temperature and fever same as the day before, as also Oct. 21, 22, 23, but continued the same treatment. Oct. 24, temperature, 103° ; Oct. 25, temperature, 102°. Let well enough alone; no change in treatment. Oct. 26, temperature, 101° ; but sick at stomach—order same pills, and substituted sulphuric acid pure for carbolic acid in 6 drops to water ℥i, teaspoonful at a dose. Oct. 27, temperature, 101°; more rational. Oct. 28, temperature, 100 ; Oct. 29, temperature, 100 ; Oct. 30, temperature, 100 ; but better bowels, all right, kidney active. Oct. 31, same. Nov. 1, same. Nov. 2, 99½ ; Nov. 3, 99; Nov. 4, 98½ ; never went to see him any more.

Mr. Monroe S——, age 30.—On the 18th December I met this gentleman, who had been to my office, but was prescribed for by another physician. Dec. 19, was called to see him. Naturally stout and robust, was suffering with a terrible headache tongue lightly coated, dark brown, centre red, tips and edges, and bowels moving constantly, thin dark, offensive matter, picking at the bed clothes and talking incoherently. Temperature 104°; pulse irregular and very frequent. Put him on the pills of opium and acet. plumbi as in the other case with same kind of stimulants and milk, as a diet. The same dose of carbolic acid, but he rejected the milk and to relieve severe gastritis, I changed to sulphuric acid, as in the case of Caleb G., with the same results.

Dec. 20, temperature, 103° ; Dec. 21, temperature, 102 ; Dec. 22, temperature, 101 ; Dec. 23, temperature, 100 ; Dec. 24, temperature, 100 ; Dec. 26, 99 ; Dec. 30, 98½. Discharged.

Mr. Robert M. S., child, 12 years of age.—Nov. 9, called to see this child with dry cough, somewhat troublesome, fever and disturbance of the bowels. Temperature, 101° ; loss of appetite, great thirst; ordered carbolic acid, one drop, and in one hour opium, gr. ⅛; acet. plumbi. gr. ¼ ; and in one hour spirits nitre 20 drops, then skip two hours and do same once again. Also ordered the bowels well rubbed with turpentine. Same stimulants and milk diet. Nov. 10, temperature, 100 ; Nov. 11, temperature 100 ; Nov. 12, 99½ ; Nov. 13, 99½ ; Nov. 14, 99½ ; Nov. 15, 99½ ; Nov. 16, 99½ ; Nov. 21, 98½. No change in treatment of this case. Discharged.

Mrs. Ramsey, age 42, very stout, weight 140.—Nov. 20, a pure case of what I designate typhoid mitior. Put this case upon the same acid treatment, and in 12 days there was no fever, but was not permitted to give the case up for 10 additional days, in which there was no fever.

Mr. Wm. Sellers, farmer, age 45, strong, and never sick before. Jan. 10, temperature, 103°. This man had been nursing at a house where several had died of typhoid fever; when he was taken sick he sent for me. He lived several miles from me and I could only see him every second or third day. But he was put on this same acid treatment, with the opiates and stimulants, with the following results: very nervous and very much alarmed from his neighbor having lost so many of his family.

Jan. 12, temperature, 102° ; Jan. 15, temperature, 101° ; Jan. 18, temperature, 100 ; Jan. 20, temperature, 99½ ; Jan. 21, temperature, 98½ ; Jan. 22, discharged. His son, a man 23 years of age, who nursed him, was also taken sick with same fever and under same treatment was clear of fever in 12 days.

Mr. David Huey, age 25, very fleshy, same symptoms ; Jan. 10, temperature, 103° ; put upon the same acid treatment; was visited every other day until Jan. 22d, when he was discharged clear of fever. Shall I enumerate any more? I can do so, but think this enough to establish the advantage of the acid treatment. I have not detailed all the symptoms, so as to convince you without a doubt that there were genuine cases of typhoid fever, but will here say that not one of them was clear of fever during the time designated by me, and several of their neighbors were sick just as they were, and continued sick under quinine treatment for thirty or forty days, and many died. But I never give quinine in this disease, and my patients recover. In these cases as supporting treatment, I made free use of cold water and ice. I had the bowels rubbed with spirits turpentine every day, and under no circumstances would I permit any of them to use solid food for days to come. Mr. A. Huey, Mr. De Waldrop, Miss A. Griffith, all cases of typhoid gravior, I have not mentioned. All treated the same way and all recovered.

